# Utility of Clinical and Ultrasonographic Hip Screening in Neonates for Developmental Dysplasia of the Hip

**DOI:** 10.7759/cureus.18516

**Published:** 2021-10-05

**Authors:** Rui Xuan Yu, Luxhman Gunaseelan, Arooj Saeed Malik, Atchaya Arulchelvan, Evan Yue, Ayesha Siddiqua, Muhammad Akhter Hamid

**Affiliations:** 1 Pediatrics, University of Toronto, Scarborough, CAN; 2 Medicine, Saba University School of Medicine, Toronto, CAN; 3 Medicine, Xavier University School of Medicine, Xavier, ABW; 4 Medicine, University of Toronto, Toronto, CAN; 5 Kinesiology, McMaster University, Hamilton, CAN; 6 Internal Medicine, Dow Medical College, Dow University of Health Sciences, Karachi, PAK; 7 Pediatrics, University of Toronto, Toronto, CAN

**Keywords:** developmental dysplasia of the hip (ddh), radiographic screening, congenital hip dysplasia, newborn screening, breech neonates, screening protocol, barlow and ortolani maneuvers, clinical screening, ultrasonographic screening, pediatric orthopedic surgery

## Abstract

Developmental dysplasia of the hip (DDH) is a musculoskeletal condition occupying any point along a spectrum of anatomical abnormalities that alter the stability of the newborn hip. Presentation varies throughout infancy and the majority of cases, especially those that are mild in nature, tend to resolve without intervention. An analysis of outcomes was conducted on infants born over a two-year period at a single-center, community hospital in East Toronto. The unwritten norm at the institution has become to order hip ultrasonography for all infants born in the breech position through C-section. Given the healthcare expenditure associated with routine radiographic screening, a careful analysis was undertaken to ascertain whether this screening regimen was effective in preventing late-stage detection of advanced DDH and improving organization in patient management. There were a total of 4236 babies delivered over the two years. One-hundred sixty-four (164) babies were born breech and through C-section. Eight (8) babies had abnormal hip examinations, one of whom was ultimately diagnosed with DDH. Forty-six (46) babies showed abnormal hip ultrasound at six weeks. Seventeen (17) referrals were made to the orthopedic surgeon. This resulted in a total of seven cases of DDH being diagnosed over the two years. The sensitivity and specificity of clinical hip screening were 14.3% and 95.5%, respectively, while that for ultrasound screening was 100% and 75.2%.

To improve the quality of care and detection of DDH, a risk factor analysis was conducted to retrospectively analyze which DDH cases would have been missed if a higher threshold to ordering hip ultrasonography had been used. Based on the test characteristics of clinical and ultrasonographic screening, held in conjunction with the risk factor analysis results, an altered screening regimen was proposed with the intention of being just as sensitive but more cost-effective. This regimen integrates clinical screening using Barlow and Ortalani maneuvers until the eight to 10-week period and examines for limited abduction from eight weeks onward. Adjuncts like the Galeazzi test and that for asymmetrical skin folds should also be included to increase the sensitivity of clinical screening. Ultrasonography is proposed for high-risk individuals, with the criteria for stratification as high-risk being extracted from the risk factor analysis. Ultrasound is also proposed to be done in a serial fashion prior to orthopedic surgery referral in cases where the age of the infant allows, which serves to better evaluate the risk for lasting DDH and understand the longitudinal trajectory of the patient. This serves the additional purpose of decreasing the psychosocial burden on families. This can be particularly significant for infants for whom the initial abnormalities are due to self-resolve with the maturation of the hip joint and the infant’s growth.

## Introduction

Developmental dysplasia of the hip (DDH) is a musculoskeletal condition occupying any point along a spectrum of anatomical abnormalities that alter the stability of the newborn hip. Clinical instability may range from mild acetabular dysplasia to total, irreducible dislocation. DDH may also manifest without clinical instability as solely radiological abnormalities [1]. The etiology is multifactorial, with the strongest risk factors being the breech position, female sex, family history, and primiparity [2-3]. Cases may present anywhere from birth to late infancy. Approximately 60-80% of hips in newborns that are clinically suspicious and 90% of those suspicious on ultrasound resolve spontaneously without intervention [4]. Nevertheless, untreated DDH can effectuate numerous morbidities, including chronic pain, degenerative joint disease, gait abnormalities, weakness, and restricted ROM. DDH is the leading cause of hip osteoarthrosis in young adults [5].

No universally accepted guidelines exist for the screening, diagnosis, and treatment of DDH. Clinical screening typically includes newborn examination via Barlow and Ortolani maneuvers. Ultrasound screening may be ordered in at-risk or suspicious cases. There is significant controversy surrounding acceptable screening protocols for DDH [6]. For a screening program to be effective, it must identify cases earlier than standard care does, and the result of the screening program should be better outcomes. The process of screening for DDH currently does not meet these criteria [4]. For starters, the effectiveness of clinical hip screening is highly examiner-dependent, and ultrasonographic screening for DDH lacks specificity [7].

The Canadian Task Force on Preventive Health Care's latest update on the screening and management of DDH in newborns stated that there is fair evidence to include serial clinical hip assessments until walking age as well as evidence to exclude universal ultrasonographic screening. They also issued low-grade recommendations against both selective and routine radiographic screening for high-risk infants [8]. The United States Preventive Services Task Force found routine screening, both clinical and radiographic, was of limited accuracy and the evidence was insufficient. They found that the clinical examination was inconsequential until later in infancy [4]. Practice guidelines from the American Academy of Pediatrics similarly recommended against universal ultrasonography, advocating for these investigations only if the infant's physical exam was inconclusive or if the infant was high-risk [9].

Attempts to develop standardized regimens for screening are further complicated by a lack of understanding of DDH’s natural history and the lack of consensus regarding which patients should be treated. There is also significant variability in treatment modality across orthopedic surgeons [6].

Overall, the management of DDH is greatly impaired by the lack of regulation and absence of guidelines surrounding screening, diagnosis, and treatment. In the absence of clear guidelines, studies are ordered according to clinician comfort. This can result in unwarranted healthcare expenditures and investigative deficiencies, wherein interpretations may fail to identify which infants are truly in need of referral and correction. An analysis was conducted, and the results are reported here on the effectiveness of screening at-risk infants with ultrasonography at a community hospital in Toronto. There is no formal hospital guideline for ordering hip ultrasonography, resulting in an unwritten standard that targets all breech-positioned infants born via C-section (BPCS). Infants born between August 2016 and July 2018 were included in the analysis, which serves to reflect on the test characteristics of clinical and radiological screening. The literature was reviewed for additional risk factors of DDH whose usage could serve to improve the screening protocol for DDH and a tailored set of screening criteria was proposed [10].

## Materials and methods

The study population comprised families that took part in the data collection. Prior to starting, approval from the ethics review board was acquired for the retrospective chart review required for the analysis. The number of newborns was collected, and they were then divided according to their method of delivery (vaginal versus C-section) and presentation (vertex versus breech). The charts were reviewed to collect the findings of their initial newborn hip assessment, which is a standard assessment conducted universally on all newborns. It was also determined from the chart whether they received an ultrasound. The results of all ultrasounds were collected on the chart. All initial ultrasounds occurred around six weeks. Repeat ultrasounds were ordered on a case-by-case basis based on radiographic certainty and the likelihood of progression to more severe disease.

Ultrasounds on the newborns were performed by radiology technicians. The results were passed along to the clinical radiologists who were responsible for reporting the findings and their impressions. Findings from the ultrasonographic hip exam included the alpha/beta angles and femoral head coverage was documented. They sometimes also included a recommendation as per whether the radiologist believed a referral to orthopedic surgery was warranted for further assessment. Diagnosis of DDH was always made by the orthopedic surgeon, who then proposes recommendations for treatment and oversees the child's care. The diagnosis and management plan were also collected from the hospital records.

To determine the utility of the screening protocols, the number of test-positive and test-negative newborns was calculated along with the number of disease-positive and disease-negative newborns. This was done for both clinical hip screening as well as for ultrasonographic screening. These numbers were used to calculate the sensitivity, specificity, positive predictive value, and negative predictive value of the screening tests. Further calculations were done to determine the time of diagnosis for cases, wherein cases were then traced back to establish how an abnormality was first identified. For newborns referred for ultrasonography, the indication provided in the initial order was identified and collected. The pre- and post-ultrasound rates of referral and diagnosis were calculated as well.

## Results

A total of 4236 babies were delivered, including 164 BPCS newborns. All babies included in the study underwent clinical hip screening performed by the pediatrician. Eight babies had abnormal newborn hip examinations. A total of 13 patient files did not include their newborn assessment data. Ultrasonography was ordered for all BPCS infants. The ultrasound screening for these newborns was performed at six weeks, and the reports showed abnormal findings in 46 babies. A total of seven newborns out of these 46 were later diagnosed with DDH by the orthopedic surgeon. Of these seven, one was initially identified as abnormal through a newborn hip exam and the remaining six were first flagged based on ultrasonography. The above information is displayed in Table 1.

**Table 1:** Study Population and Results of Clinical and Ultrasonographic Screening C/S: C-section; US: Ultrasound screening; DDH: developmental dysplasia of the hip A total of 4236 neonates were delivered over the two-year period, 1488 of which were born via C-section. One-hundred sixty-four (164) of these C-sections were done due to the babies being in breech position. Eight (8) infants showed abnormal clinical screening and 13 patient files did not include the results of the newborn assessment. Forty-six (46) infants had an abnormal ultrasound. Seven (7) of these infants went on to receive a diagnosis of DDH.

Total Number of Deliveries	Total Number of C/S	Total Number With Breech Presentation	Number of Abnormal Newborn Assessment	Number of Normal Newborn Assessment	Number of Babies With Missing NB Data
4236	1488	164	8	143	13
Abnormal Newborn Assessment			Normal or No Newborn Assessment		
Abnormal US	Normal US	No US	Abnormal US	Normal US	No US
1	7	0	45	111	0
7			Final Diagnosis of DDH		

At this hospital, infants that are likely to be in breech position on their due date are recommended to be born via C-section to decrease the potential for complications in both the mother and baby. However, as there is an increased risk for hip abnormalities in BPCS infants, most clinicians elect to uniformly order hip ultrasonography for these infants regardless of the ease of delivery or additional risk factors. All seven cases of DDH showed hip abnormalities on ultrasound, rendering the sensitivity of ultrasound screening to be 100%. The specificity was much lower at 75.2%. For clinical screening of the eight infants who initially showed abnormalities on the newborn hip assessment, only one of these eight received an eventual diagnosis of DDH. The sensitivity of clinical DDH screening was 14.3% and the specificity was 95.5%. The test characteristics of both clinical and ultrasonographic screening are shown in Table 2.

**Table 2:** Test Characteristics for Clinical and Ultrasound DDH Screening DDH: developmental dysplasia of the hip

Clinical Exam	Disease +		Disease -	Totals	Ultrasound		Disease +		Disease -	Totals
Test +	1	7		8	Test +	7		39		46
Test -	6		150	156	Test -	0		118		118
Totals	7		157		Totals	7		157		
Sensitivity			.143		Sensitivity			1		
Specificity			.955		Specificity			.752		
Positive Predictive Value			.125		Positive Predictive Value			.152		
Negative Predictive Value			.962		Negative Predictive Value			1		
False Positive Rate			.045		False Positive Rate			.248		
False Negative Rate			.857		False Negative Rate			0		

Of the 46 abnormal ultrasounds, 17 were referred to the orthopedic surgeon based on the combined clinical judgment of the pediatrician and radiologist. It was the responsibility of the pediatrician to refer infants for whom they were concerned to orthopedic surgery for further assessment. However, the radiologist would often indicate their own concerns in their report, which might prompt the pediatrician to refer those infants more urgently. The pre-test orthopedic surgery referral rate for breech infants born via C-section was 10.4%. If any abnormality was found on ultrasound, this percentage increased to 40.0%. The remainder of BPCS infants either showed no ultrasonographic abnormalities or showed abnormalities that resolved without referral or intervention. The incidence of DDH within the BPCS population of infants was 4.3%. The positive predictive value for hip ultrasonography was 15.2%, meaning that of 100 babies receiving a positive ultrasound result, 15 of them would eventually receive a diagnosis of DDH. Approximately a quarter of all initial ultrasounds were followed by a repeat ultrasound at 10-12 weeks of age for reassessment. The decision for a repeat ultrasound was made by the pediatrician, often under the recommendation of the radiologist. The repeat ultrasound could also be ordered by the orthopedic surgeon after receiving the referral. There was no standard protocol for repeat hip ultrasonography at this hospital. The average age of diagnosis after ultrasonography was six to 12 weeks. The primary treatment for babies with DDH was the Pavlik harness.

## Discussion

Children with clinical instability were diagnosed significantly younger than those diagnosed after hip ultrasonography. The diagnosis was made at one to two weeks in the former and six to 12 weeks in the latter. The utility of earlier diagnosis depends on the nature and effectiveness of the early intervention. Earlier diagnoses may be beneficial in more severe cases of dysplasia, wherein clinical detectability may positively correlate with that increased severity. In cases where there is suspicion for a high-grade dysplasia evident on clinical assessment, ultrasonographic investigations could be considered immediately rather than as screening at six weeks of age. At present, there is no conclusive evidence for such expedited diagnosis, and more research is needed to determine when benefits are conferred.

The ostensible sensitivity of current clinical DDH screening is alarmingly low. Seven babies were diagnosed with only one showing initial clinical abnormalities. This is likely attributable to multiple factors. Around 200 babies are born each month and generally assessed by the pediatrician on call. The sheer volume of births results in follow-up for any specific clinician potentially being inadequate for a good clinical correlation of clinical findings and ultrasonographic detection. Additionally, the “safety net” of six-week ultrasounds and lack of evidence favoring earlier diagnosis may decrease the duty and necessity to conduct a thorough hip examination. Furthermore, the continual growth occurring in infants means that there could be delays in the clinical manifestation of hip abnormalities and changes thereafter, thereby evading detection. Often, DDH is undetectable at birth but presents in the first few months with growth and movement [11]. Yang et al. found a sensitivity of 54% for the Barlow and Ortolani maneuvers while Sulaiman et al. found the sensitivity to be 66.7% when these tests were performed by a trained examiner, with the sensitivity of the tests increasing for experienced clinicians [7,12]. In our study, the sensitivity was 14.3%, which may indicate a lack of longitudinal follow-up examinations or that more training is needed when it comes to clinical signs. The incidence of DDH was only 0.16%, wherein this low rate might inspire a low baseline clinical suspicion and an element of confirmation bias. This could subconsciously mask mild findings, especially when most hip sounds and initial dysplasia will self-resolve as the child grows. Given the exam’s poor sensitivity, adjunct clinical examinations may be of benefit to increase detection. Current research supports improved detection when additional maneuvers are added [7].

Ultrasonography had a positive predictive value of 15.2%, meaning that a large majority of the positive tests did not result in a diagnosis of DDH. It is important to consider the intangible losses that false positives cause. False positives arouse concern in the patient’s family, often with extensive time spent worrying, for appointments, and testing. 17 of 46 patients were referred to orthopedic surgery, with the pre- and post-test referral rates being 10.4% and 37.0%, respectively. The approximate priority 3 wait time for pediatric orthopedic surgery in Ontario is 64 days [13]. Even so, this correlates to 39 concerned families whose children never developed DDH, 640 days of waiting for specialist appointments with no eventual diagnosis, and many more hundreds of dollars of resources used for appointments and diagnostic imaging.

Given the wait time for orthopedic consultation and the rate of spontaneous resolution, it may be advisable from a resource stewardship standpoint to practice a period of watchful waiting, with or without additional ultrasonographic investigations prior to referral. The first hip ultrasound occurs at six weeks or when clinicians have increased suspicion for dysplasia. A second ultrasound at around 12 weeks could be considered prior to the infant's referral to orthopedic surgery. DDH discovered prior to six months is generally treated conservatively with a Pavlik harness [12]. Instituting recommendations for watchful waiting periods may also be beneficial given the poor PPV of ultrasonographic hip assessment. This observatory period can be more cost-effective and prevent overtreatment, which may benefit the family and patient by reducing hospital visits and psychological repercussions within the treatment process [14].

Our proposed modifications to the current approach center around improving the sensitivity of the clinical exam through altering the methodology and implementing them in a serial fashion, using serial ultrasounds, and increasing the clarity of radiographic reports. Providing education regarding the utilization of the appropriate maneuvers for an infant's age could improve exam detection and accuracy. The Barlow and Ortolani maneuvers are possible at birth because of the hip joint’s ligamentous hyperlaxity [11]. However, clinicians may be unaware that within several weeks, the hyperlaxity resolves, thus reducing the accuracy of these two maneuvers. By two months, the preferred method is assessment for limited hip abduction. An improved clinical screening protocol may involve Barlow and Ortolani maneuvers performed at birth and six weeks with the integration of adjuncts like an inspection for asymmetric skin folds, Galeazzi's test, and limb length discrepancies. Starting at eight to 10 weeks, infants can also be examined for limited abduction. A clinical examination could occur when patients come in for hip ultrasonography to increase patient convenience and reduce unnecessary costs. Education could occur not only for pediatricians but also for family doctors so that ideally, every infant has at least three data points in the way of clinical hip assessment using the best test for their age.

There may also be an advantage to decreasing the number of ultrasounds ordered for BPCS infants. Over the period of two years, there were seven cases of DDH discovered and treated at our single center. According to the Cochrane review, some significant risk factors in DDH include primiparity, female sex, race, family history, postmaturity, torticollis, foot abnormalities, such as talipes or metatarsus adductus, and clinical instability [2]. There was no data to support increased DDH prevalence in a specific demographic within Toronto, though general literature shows a higher prevalence in Caucasian and first-nations groups [10]. A retrospective chart review was done to identify risk factors present in each of the seven children who were diagnosed with and treated for DDH during the span of two years. We looked at nine additional risk factors: clinical instability, female sex, primiparity, postmaturity, family history, ethnic background, oligohydramnios, torticollis, and foot abnormalities [2]. All seven babies had at least one risk factor aside from being breech C-sections, ranging from 1/9 to 4/9 of these additional risk factors. Results of the risk factor analysis are shown in Table 3.

**Table 3:** Risk Factor Analysis of Diagnosed DDH Cases Of the seven cases, 6/7 were female and 5/7 were firstborn children. 2/7 of the cases showed clinical instability on examination: one of these was during the newborn examination while the other was at five weeks. The former was referred for immediate ultrasonography while the latter child was already scheduled for a six-week ultrasound so the management did not change. 2/7 had a relevant family history, wherein one was a sibling with DDH while the other was a mother with hip arthritis. 2/7 were of Caucasian or first-nations descent. 2/7 were diagnosed with comorbid torticollis. None of the babies were postmature or showed any foot abnormalities. DDH: developmental dysplasia of the hip

DDH Cases	Case 1	Case 2	Case 3	Case 4	Case 5	Case 6	Case 7	Total
Ultrasound								7/7
Breech C/S								7/7
Risk Factors								
Clinical	Newborn				5 weeks			2/7
Female								6/7
Firstborn								5/7
Postmature	39+w	38+w	38+w	38+w	39+w	36+w	38+w	0/7
Caucasian/First Nations								2/7
­­Family Hx	DDH				Arthritis			2/7
Torticollis								2/7
Foot Abns								0/7
Oligohydramnios								0/7
Total RFs	4/9	3/9	1/9	1/9	4/9	2/9	4/9	

Potential criteria for a more targeted set of criteria for ultrasonographic screening of BPCS infants was devised based on the risk factor analysis, wherein ultrasonographic screening would only be ordered if infants are deemed high-risk based on the following criteria:

The infant is born via BPCS and one or more of the following:

1. Abnormal newborn hip exam; OR

2. Any one or more of the following risk factors: female, family history, firstborn, postmaturity, Caucasian or first-nations ethnic background, oligohydramnios, torticollis, foot abnormalities; OR

3. There is uncertainty regarding the presence of risk factors; OR

4. The clinician is unable to satisfactorily complete the clinical hip exam; OR

5. The delivery was complicated and there is a concern for stress on the infant's hips.

This way, rather than blanketing all BPCS infants, high-risk infants could instead be identified as those with at least one other concerning factor in the infants' history and physical exam. This could be a factor in history or be a finding on a physical exam. Infants meeting this requirement should continue to receive hip ultrasonography. Serial ultrasound could be considered for longitudinal correlation, to determine whether dysplasias will resolve or worsen over time. Cases that are unlikely to resolve would continue to be referred. So long as both ultrasounds and the consult take place prior to six months of age, the treatment of the infant is unlikely to change. Additionally, since the vast majority of potential dysplasias and joint irregularities will self-resolve [4], delaying referral, especially in cases where of mild ultrasonographic abnormality, may be effective in reducing unnecessary intervention and reducing healthcare costs.

In terms of ultrasonography reporting, unifying the ultrasound technique and reporting method may allow for better stratification of dysplastic severity. Graf’s static assessment of hip morphology emphasizes technique and congruent interpretation styles in ultrasound analysis. Although it remains limited by operator-dependent factors, uniform utilization of the Graf method to communicate findings may be effective for standardizing findings [15]. This will also make the recommended observational period much clearer. Ultimately, more informative and prescriptive impressions from radiology may allow for more concrete management plans.

For individuals who are diagnosed with DDH, we identified deficiencies in their management plan pertaining to follow-up. Oftentimes, patients that were diagnosed with DDH were not followed up to assess treatment effectiveness. Although repeat ultrasounds may not be necessary, it would be useful to query the state of the patient’s hips. The upsurge in usage and acceptability of virtual appointments could allow doctors to improve follow-up, wherein observations and outcomes could be elicited over audio or video calls. Even in the absence of worrisome signs and symptoms, adequate follow-up could foster a sense of closure and provide better documentation for quality improvement (QI) purposes.

In summary, we propose an integrated clinical and ultrasonographic approach for the optimal detection of DDH. This approach would integrate serial clinical examinations throughout infancy at well-baby visits as well as additional assessments based on the clinician’s impression. Ultrasound of the hips would occur for high-risk individuals, identified as above, at the six and 12-week marks. Standardizing radiographic reports and recommendations for specific observational periods will allow for risk stratification and resource stewardship. By correlating radiographic impressions with the clinical circumstances, patients who are unlikely to self-resolve or for whom there is significant uncertainty could be referred for specialist assessment. Reducing the number of patients referred also expedites assessment and management for those needing it. Follow-up should occur after the intervention to assess the effectiveness and gather data for QI.

A limitation in our current study is that our methodology would not have captured missed cases of DDH that were cared for in other centers. We plan on expanding this study to include follow-up from other centers, to better discern the utility of clinical and ultrasonographic hip screening in neonates for DDH.

Ultimately, there is still much we are in the dark about when it comes to DDH. In 1968, the WHO identified criteria that good screening programs would satisfy, including conditions on disease significance, accepted treatment protocols, and test characteristics [16]. Although DDH meets the WHO criteria for disease significance and importance, it falls short on its treatments and the test characteristics for both clinical and ultrasonographic screening make it difficult to justifies the costs to the healthcare system. Without any widely accepted treatment protocol or consensus on which patients should be treated, the intended management and outcome that is to result from DDH screening becomes uncertain, which is additionally confounded by our inability to predict which cases will self-resolve. In the absence of a latent or early-symptomatic stage and a thorough understanding of DDH’s natural history, the intention and potential of screening remain unclear.

Overall, more research is needed on DDH as a disease, particularly on its natural history, and to identify tools for stratification of cases to determine who is likely to benefit from early intervention. Once there is further knowledge on what features would warrant concern and treatment, a screening regimen can be better designed to improve disease management and patient outcomes.

## Conclusions

Our study demonstrates the shortcomings of both clinical and ultrasonographic screening modalities for DDH. Ultrasonographic screening for all BPCS infants demonstrated inadequacy in ruling out DDH, thereby increasing the risk for both tangible repercussions, in the way of healthcare expenditures, and intangible consequences, in the way of familial burden. Because so many initial abnormalities in infants’ hips are self-limited, ultrasonographic screening in infants that are not truly high-risk runs the risk of wasting significant healthcare resources and inspiring emotional distress without reason in many families. Ultrasonographic screening did detect DDH cases earlier than clinical screening, but this early discovery does not necessarily improve patient outcomes. On the flip side, clinical screening is lighter on resource usage, however, it falls short in terms of sensitivity of DDH detection - particularly in higher-risk groups. To attenuate this imbalance, the first step may be redefining which infants we consider to be truly high risk. Thereby, in confining the number of hip ultrasounds ordered for a population for which the prevalence is higher, there would be a correlated decrease in the rate of false positives, thereby improving the positive predictive value. In the way of improving the sensitivity of clinical screening, there may be a benefit to improving the knowledge regarding screening maneuvers in the clinician population, given the strong dependence of their utility on user expertise. Improvements could also be made in the way of longitudinal correlation and improving clinicians' clinical exposure to known DDH cases, which would improve the detection rate of infants that are clinically abnormal on a newborn hip exam.

Neither clinical nor ultrasonographic screening meets the requirements of an effective screening program by themselves, but through careful combination, they may be able to detect cases of DDH earlier and more effectively, without such excessive testing in lower-risk populations. We propose that this be done through changing the criteria for ultrasonographic screening, improving education surrounding clinical maneuvers, longitudinal correlation of clinical findings in infants prior to referral, as well as standardizing radiographic techniques and reporting. We hope that these changes will decrease healthcare resource expenditure without decreasing the detection rate of DDH. More research is needed to expand this analysis to larger centers as well as to better understand the characteristics and optimal treatment of DDH as a disease.

## References

[1] Dezateux C, Rosendahl K (2007). Developmental dysplasia of the hip. Lancet.

[2] Shorter D, Hong T, Osborn DA (2013). Cochrane Review: screening programmes for developmental dysplasia of the hip in newborn infants. Evid Based Child Health.

[3] Kural B, Devecioğlu Karapınar E, Yılmazbaş P, Eren T, Gökçay G (2019). Risk factor assessment and a ten-year experience of DDH screening in a well-child population. Biomed Res Int.

[4] US Preventive Services Task Force (2006). Screening for developmental dysplasia of the hip: recommendation statement. Pediatrics.

[5] Al-Essa RS, Aljahdali FH, Alkhilaiwi RM, Philip W, Jawadi AH, Khoshhal KI (2017). Diagnosis and treatment of developmental dysplasia of the hip: a current practice of paediatric orthopaedic surgeons. J Orthop Surg (Hong Kong).

[6] Paton RW (2017). Screening in developmental dysplasia of the hip (DDH). Surgeon.

[7] Sulaiman A, Yusof Z, Munajat I, Lee N, Zaki N (2011). Developmental dysplasia of hip screening using Ortolani and Barlow testing on breech delivered neonates. Malays Orthop J.

[8] Patel H (2001). Preventive health care, 2001 update: screening and management of developmental dysplasia of the hip in newborns. CMAJ.

[9] American Academy of Physicians (2017). Developmental dysplasia of the hip in infants: a clinical report from the AAP on evaluation and referral. Am Fam Physician.

[10] Loder RT, Skopelja EN (2011). The epidemiology and demographics of hip dysplasia. ISRN Orthop.

[11] Jackson JC, Runge MM, Nye NS (2014). Common questions about developmental dysplasia of the hip. Am Fam Physician.

[12] Yang S, Zusman N, Lieberman E, Goldstein RY (2019). Developmental dysplasia of the hip. Pediatrics.

[13] Health Quality Ontario (2021). Health Quality Ontario. Time to pediatric patient’s first surgical appointment. https://www.hqontario.ca/System-Performance/Wait-Times-for-Surgeries-and-Procedures/Pediatric-Wait-Times-for-Surgeries/Time-to-Pediatric-Patients-First-Surgical-Appointment.

[14] Gardner F, Dezateux C, Elbourne D, Gray A, King A, Quinn A (2005). The hip trial: psychosocial consequences for mothers of using ultrasound to manage infants with developmental hip dysplasia. Arch Dis Child Fetal Neonatal Ed.

[15] Jacobino BC, Galvão MD, da Silva AF, de Castro CC (2012). Using the Graf method of ultrasound examination to classify hip dysplasia in neonates. Autops Case Rep.

[16] Wilson JMG, Jungner G (1968). Principles and practice of screening for disease. World Health Organization: Public Health Papers.

